# Complete genome sequence of *Pyrobaculum oguniense*

**DOI:** 10.4056/sigs.2645906

**Published:** 2012-07-20

**Authors:** David L. Bernick, Kevin Karplus, Lauren M. Lui, Joanna K. C. Coker, Julie N. Murphy, Patricia P. Chan, Aaron E. Cozen, Todd M. Lowe

**Affiliations:** 1 Biomolecular Engineering, University of California., Santa Cruz, California, USA

**Keywords:** *Pyrobaculum oguniense*, *Pyrobaculum arsenaticum*, *Crenarchaea*, inversion

## Abstract

*Pyrobaculum oguniense* TE7 is an aerobic hyperthermophilic crenarchaeon isolated from a hot spring in Japan. Here we describe its main chromosome of 2,436,033 bp, with three large-scale inversions and an extra-chromosomal element of 16,887 bp. We have annotated 2,800 protein-coding genes and 145 RNA genes in this genome, including nine H/ACA-like small RNA, 83 predicted C/D box small RNA, and 47 transfer RNA genes. Comparative analyses with the closest known relative, the anaerobe *Pyrobaculum arsenaticum* from Italy, reveals unexpectedly high synteny and nucleotide identity between these two geographically distant species. Deep sequencing of a mixture of genomic DNA from multiple cells has illuminated some of the genome dynamics potentially shared with other species in this genus.

## Introduction

*Pyrobaculum oguniense* TE7^T^ (=DSMZ 13380=JCM10595) was originally isolated from the Tsuetate hot spring in Oguni-cho, Kumamoto Prefecture, Japan [1], and subsequently found to grow heterotrophically at an optimal temperature near 94°C, pH 7.0 (at 25°C), and in the presence or absence of oxygen. Under anaerobic conditions, it can utilize sulfur-containing compounds (sulfur, thiosulfate, L-cystine and oxidized glutathione**)** but not nitrate or nitrite as terminal electron acceptors.

Initial 16S ribosomal DNA sequence analysis [1] placed *Pyrobaculum oguniense* TE7^T^ in the *Pyrobaculum* clade and closest to *P. aerophilum* and *Thermoproteus neutrophilus* (now considered a member of the genus *Pyrobaculum* [50]). DNA hybridization studies were conducted with *P. aerophilum* IM2, *P. islandicum* geo3, *P. organotrophum* H10 and *T. neutrophilus* V24Sta, showing little genomic similarity to those species. *P. arsenaticum* PZ6^T^ [2] , *P.* sp.1860 [3] and *P. calidifontis* VA1 [4] were not available at that time.

The genus *Pyrobaculum* is known for its range of respiratory capabilities [5]. Three of the currently known members of the genus can respire oxygen; *P. aerophilum* is a facultative micro-aerobe, while *P. calidifontis* and *P. oguniense* can utilize atmospheric oxygen. *P. aerophilum* [6], *P. calidifontis*, and four other metabolically unique *Pyrobaculum* species have been fully sequenced; together with *P. oguniense*, we sought to further broaden the understanding of this important hyperthermophilic group.

Pairwise whole-genome alignments of previously sequenced *Pyrobaculum* species reveal many structural rearrangements. With the availability of high-throughput sequencing, we were able to further explore rearrangements that occur between species, and our use of a not-quite-clonal population allowed exploration of rearrangements within a single species.

## Classification and features

**Figure 1:**
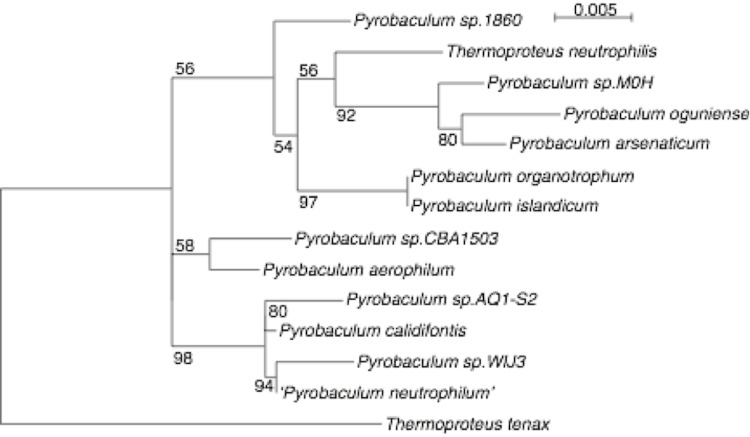
Phylogenetic tree of the known *Pyrobaculum* species based on 16S ribosomal RNA sequence. Accession numbers and associated culture collection identifiers (when available) for 16S ribosomal RNA genes are: *Pyrobaculum aerophilum* (NC_003364.1, DSM 7523); *P. calidifontis* (NC_009073.1, DSM 21063); *P. islandicum* (NC_008701.1, DSM 4184); *P. arsenaticum* (NC_009376.1, DSM 13514); *P. oguniense* (CP003316, DSM 13380); *Thermoproteus neutrophilus* (NC_010525.1, DSM 2338); *P.sp.1860* (CP003098.1); *P. organotrophum* (AB304846.1, DSM 4185); *P.sp.CBA1503* (HM594679.1); *P.sp.M0H* (AB302407.1); *P.sp.AQ1.S2* (DQ778007.1); *P.WIJ3* (AJ277125.1); *‘P. neutrophilum’ (X81886)*. Sequences were aligned using MAFFT v.6 [7], followed by manual curation [8] to remove 16S ribosomal introns and all terminal gap columns caused by missing sequence. The maximum likelihood tree was constructed using Tree-Puzzle v. 5.2 [9] using exact parameter estimates, 10,000 quartets and 1000 puzzling steps. *Thermoproteus tenax Kra1* (NC_016070.1, DSM 2078) was included as an outgroup. Numbered branches show bootstrap percentages and branch lengths depict nucleotide mutation rate (see scale bar upper right).

Figure 1 and Table 1 summarize the phylogenetic position and characteristics of *Pyrobaculum oguniense* TE7 relative to other members of the *Pyrobaculum* genus, respectively.

**Table 1:** Classification and general features of *Pyrobaculum oguniense* according to the MIGS recommendations [10].

**MIGS ID**	**Property**	**Term**	**Evidence code**^a^
	Current classification	Domain *Archaea*	TAS [11]
		Phylum *Crenarchaeota*	TAS [12]
		Class *Thermoprotei*	TAS [13,14]
		Order *Thermoproteales*	TAS [15-18]
		Family *Thermoproteaceae*	TAS [15-17]
		Genus *Pyrobaculum*	TAS [19,20]
		Species *Pyrobaculum oguniense*	TAS [1]
		Type strain *TE7*	
	Cell shape	rods 0.6-1µm × 2-10µm	TAS [1]
	Motility		
	Sporulation	no	
	Temperature range	70–97°C	
	Optimum temperature	90–94°C	
	Carbon source	heterotroph1g/L yeast extract or 0.5g/L yeast extract with 0.5g/L tryptone)	TAS [1]
	Energy source	(see carbon source)	TAS [1]
	Terminal electron acceptor	O_2_, sulfur compounds, no growth on NO_3_ or NO_2_	TAS [1]
MIGS-6	Habitat	hot-spring	TAS [1]
MIGS-6.3	Salinity	0–1.5% (w/v); 0% optimal	TAS [1]
MIGS-22	Oxygen	facultative aerobe	TAS [1]
MIGS-15	Biotic relationship	free-living	NAS
MIGS-14	Pathogenicity	none	NAS
MIGS-4	Geographic location	Tsuetate hot spring, Oguni-cho, Kumamoto prefecture, Japan	TAS [1]
MIGS-5	Sample collection time	June 1997	NAS
MIGS-4.1	Latitude	33.186	NAS
MIGS-4.2	Longitude	131.031	NAS
MIGS-4.3	Depth	hot-spring sediment / fluid	NAS
MIGS-4.4	Altitude	300m	NAS

Evidence codes - TAS: Traceable Author Statement; NAS: Non-traceable Author Statement. These evidence codes are from the Gene Ontology project [22].

## Genome sequencing information

### Genome project history

Table 2 presents the project information and its association with MIGS version 2.0 compliance [10].

**Table 2:** Project information

**MIGS ID**	**Property**	**Term**
MIGS-31	Finishing quality	Finished
MIGS-28	Libraries used	Roche 454 Titanium library, SOLiD 2×25 Mate-pair (1k-3.5k insert)
MIGS-29	Sequencing platforms	454 GS FLX Titanium, ABI SOLiD
MIGS-31.2	Fold coverage	59× 454, 500× SOLiD
MIGS-30	Assemblers	Newbler 2.0.01.14, Custom
MIGS-32	Gene calling method	Prodigal, tRNAScan-SE
	Genome Database release	Genbank
	Genbank ID	379005763 379002962
	Genbank Date of Release	2012-02-12
	GOLD ID	Gi05801
	Project relevance	Biotechnology

### Growth conditions and DNA isolation

The initial culture was obtained in 2003 from the *Leibniz Institute-German Collection of Microorganisms and Cell Cultures* (DSMZ), and grown anaerobically in stoppered, 150ml glass culture bottles at 90°C. This culture was stored at 4°C for an extended period (six years) before being sampled for this study.

A set of ten-fold dilutions of an actively growing culture (~10^8^ cells/ml) was carried out and growth was monitored over a five-day period. All cultures were grown at 90°C without shaking in 200ml modified DSM 390 medium, using 1g tryptone, 1g yeast extract, pH 7, supplemented with 10mm Na_2_S_2_O_3_ in 1L flasks under a headspace of nitrogen. At day four of growth, a new 400ml aerobic culture was inoculated with 20ml from the penultimate member of the dilution series (10^-8^) and shaken at 100 rpm, supplemented with 10mM Na_2_S_2_O_3_, and subsequently was used for sequencing. We note that at day five, turbid growth was seen in the final member of the dilution series (10^-9^ initial dilution). This implies that the initial 10^-8^ inoculum used for sequencing likely included more than 10 cells.

Cell pellets were obtained from the 400ml aerobic culture, frozen at -80°C and suspended in 15ml SNET II lysis buffer (20mM Tris-Cl pH 8, 5mM EDTA, 400mM NaCl, 1% SDS) supplemented with 0.5mg/ml Proteinase K and incubated at 55°C for four hours. DNA was extracted from this digest using an equal volume of Tris-buffered (pH 8) PCI (Phenol:Chloroform:Isoamyl-OH (25:24:1)). Following phase-separation (3220g, 10 min. at 4°C), the resulting aqueous phase was treated with RNase A (25µg/ml) for 30 minutes at 37°C. This reaction was PCI-extracted a second time, followed by CHCl_3_ extraction of the resulting aqueous phase and a final phase separation as before.

DNA was precipitated in an equal volume of isopropyl alcohol at -20°C overnight, followed by centrifugation (3,220 g, 15 min. at 4°C). The resulting pellet was washed in 70% EtOH, pelleted (3220g, 30 min. at 4°C) and aspirated to remove the supernatant. The final DNA pellet was suspended in 1ml TE (50mM Tris-Cl Ph 8, 1 mM EDTA) overnight at room temperature, yielding a final DNA concentration of 0.77 µg/µl.

### Genome sequencing and assembly

Sequencing was performed by the UCSC genome sequencing center using both Roche/454 GS/FLX Titanium pyrosequencing and the ABI SOLiD system (mate-pair). Pyrosequencing reads were assembled with 59X coverage exceeding Q40 over 99.95% (2,449,310 bases) of the genome, producing 20 contigs at an N50 of 467,815 bp. This assembly included 24 Sanger reads generated by primer-walking across four of the five encoded CRISPR repeat regions. The resulting maximal base-error rate (<Q40) is 25 in 50,000.

Contigs were assembled to a single scaffold using the mate-pair library generated for use on the ABI SOLiD sequencer. The library was produced with an insert size range of 1000–3,500 bp, and final sequencing yielded 30,631,205 read pairs of 25 bp read length. Those read-pairs were mapped to the 20 pyrosequencing-derived contigs to produce a *From::To* table of uniquely mapping read-pairs; accumulated for each of the 20×20 contig-pair assignments in each of the three possible relative contig orientations (same, converging or diverging). The scaffold closed easily with these data and yielded a single main chromosome with three major inversions and an extra-chromosomal element.

### Genome annotation

Gene prediction and annotation was prepared using the IMG/ER service of the Joint Genome Institute [25], where protein coding genes were identified using Prodigal [26] RNase P RNA [27], SRP RNA and ribosomal RNA(5S, 16S, 23S) were identified by homology to the currently described *Pyrobaculum* members using the UCSC Archaeal Genome Browser (archaea.ucsc.edu) [28]. Annotation of transfer RNA (tRNA) genes was established using tRNAscan-SE [29], supplemented with manual curation of non-canonical introns. C/D box sRNA genes were identified computationally using Snoscan [30] with extensions supported by transcriptional sequencing [51]. H/ACA-like sRNA genes were identified using transcriptionally-supported homology modeling of experimentally validated sRNA transcripts [31]. CRISPR repeats were identified using CRT [32] or CRISPR-finder [33], with strandedness established by transcriptional sequencing.

## Genome properties

The properties and overall statistics of the genome are summarized in Table 3, Table 4, Table 5, Table 6, and Table 7. The single main chromosome (55.08% GC content) has a total size of 2,436,033 bp. Ultra-deep mate-pair sequencing has revealed three regions of the genome that are present in an inverted orientation within a minority of the population (Table 7). The genome also includes an extra-chromosomal element of 16, 887 bp (50.58% GC), that encodes 35 predicted protein-coding genes. Of those genes, seven have an annotated function and the remaining 28 genes are annotated as hypothetical proteins. Of the seven annotated genes, three are coded with viral functions [34]. 

**Table 3:** Nucleotide content and gene count levels of the main chromosome^a^

**Attribute**	**Value**	**% of total**
Genome size (bp)	243,6033	100
DNA Coding region (bp)	2,164,251	88.84
DNA G+C content (bp)	1,341,816	55.08
Total genes	2,980	100
RNA genes	145	4.74
rRNA operons	1	
Protein-coding genes	2,800	93.96
Genes in paralog clusters	1,214	40.74
Genes assigned to COGs	1,797	60.30
Genes assigned PFAM domains	1,719	57.68
Genes with signal peptides	794	26.64
Genes with transmembrane helices	646	21.68
CRISPR arrays	5	% of total

^a^The ECE (16,887 bp) contains 35 genes, has a 50.58% G+C content, and is excluded from this table. Total gene count includes 35 pseudogenes.

**Table 4:** Number of genes associated with the 25 general COG functional categories

**Code**	**Value**	**%age^a^**	**Description**
J	163	8.53	Translation
A	5	0.26	RNA processing and modification
K	112	5.86	Transcription
L	100	5.23	Replication, recombination and repair
B	4	0.21	Chromatin structure and dynamics
D	22	1.15	Cell cycle control, mitosis and meiosis
Y	NA		Nuclear structure
V	15	0.78	Defense mechanisms
T	45	2.35	Signal transduction mechanisms
M	47	2.46	Cell wall/membrane biogenesis
N	4	0.21	Cell motility
Z	1	0.05	Cytoskeleton
W	NA		Extracellular structures
U	22	1.15	Intracellular trafficking and secretion
O	87	4.55	Post-translational modification, protein turnover, chaperones
C	182	9.52	Energy production and conversion
G	82	4.29	Carbohydrate transport and metabolism
E	159	8.32	Amino acid transport and metabolism
F	58	3.04	Nucleotide transport and metabolism
H	115	6.02	Coenzyme transport and metabolism
I	60	3.14	Lipid transport and metabolism
P	83	4.34	Inorganic ion transport and metabolism
Q	26	1.36	Secondary metabolites biosynthesis, transport and catabolism
R	323	16.90	General function prediction only
S	196	10.26	Function unknown
-	1144		Not in COGs

**Table 5:** Sixteen largest regions present in *Pyrobaculum oguniense* and absent in *P. arsenaticum.*

**Region coordinates (kb)**	**PaRep type**	**Gene cluster**
2,420 - 0,020	paREP2	
420 - 440	paREP1/8	
485 - 530	paREP2	
682 - 695	paREP2	
887 - 900		ThiW
955 - 985	paREP1/8	CRISPR cassette
1,090 - 1,120	paREP1	Cobalamin biosynthesis cassette
1,160 - 1,180		CO dehydrogenase
1,235 - 1,250	paREP1/8	
1,440 - 1, 460	paREP1/8	
1,540 - 1,565		aerobic terminal cytochromes
1,672 - 1,690	paREP6	
1,715 - 1,735		CO dehydrogenase
1,780 - 1,795	paREP1	
1,825 - 1,870	paREP2	
2,300 - 2,385		ThiC

**Table 6:** Summary of genome: one chromosome and one extra-chromosomal element

Label	Size (bp)	Topology	INSDC identifier
Chromosome (Chr)	2,436,033	circular	NC_016885.1
Extra-chromosomal Element (ECE)	16,887	circular	NC_016886.1

**Table 7:** Genomic inversions present within the sampled population

Inversion name	Coordinates			
	**Start**	**End**	**Length**	**Frequency**
GluDH	50,930	223,540	172,611	0.17
RAMP/paREP	932,090	955,719	23,630	0.18
C8	1,686,376	1,708,299	21,924	0.35

^a^Minority inversion frequency established as described previously [24].

^a^The total is based on the 1,911 COG assignments made across 1,701 protein-coding genes with at least one COG assignment. The *Not in COGs* category is made up of 1,099 hypothetical protein coding genes and 145 RNA genes. The 35 genes in the ECE are excluded from this analysis.

**Figure 2:**
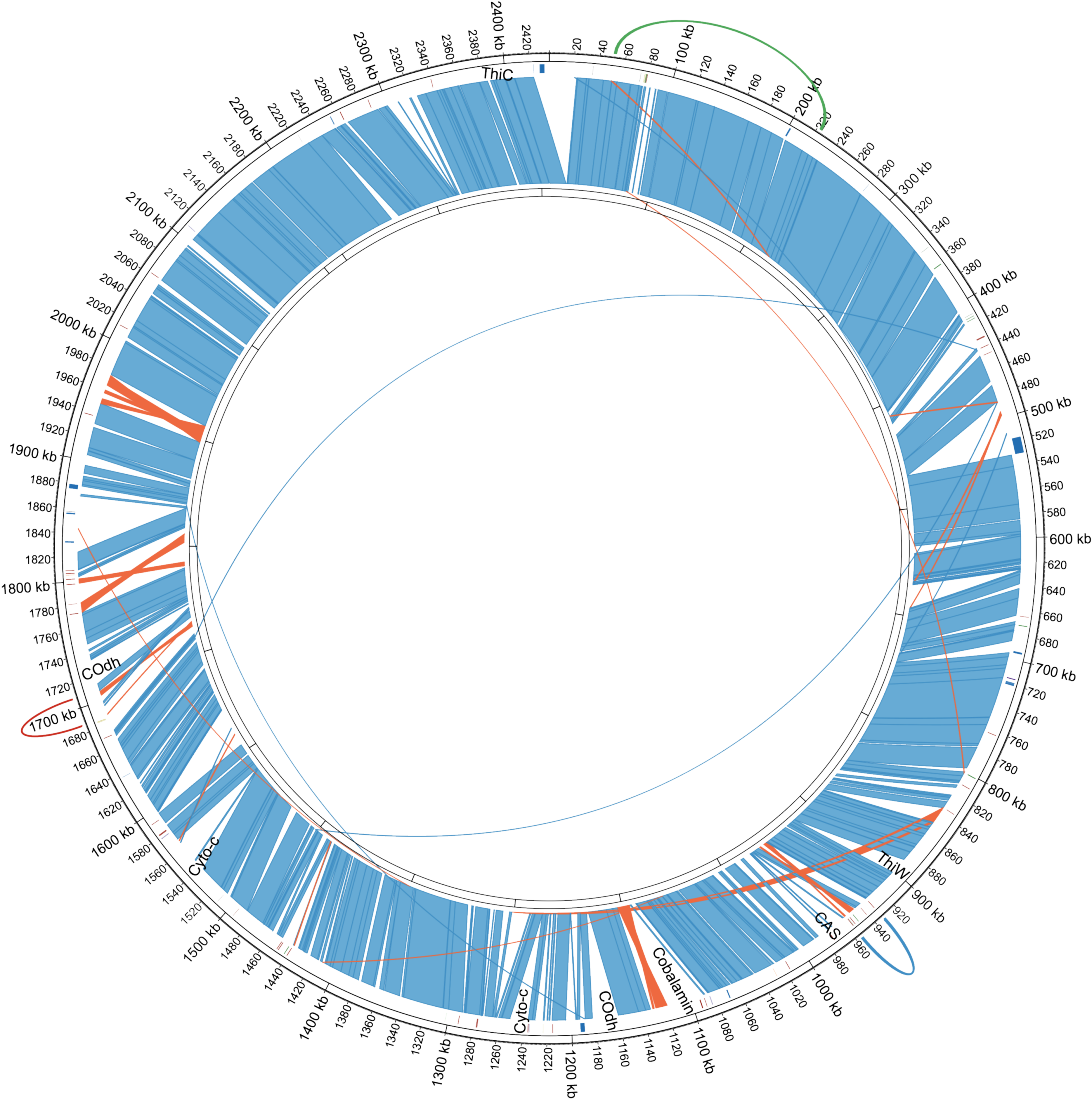
Genomic alignment of *P. oguniense* with *P. arsenaticum*. Outer ring: *P. oguniense* (+ strand); Inner ring: *P. arsenaticum* (- strand). Inter-species alignment blocks shown in light blue and gold (inverted orientation). Intra-species *P. oguniense* genomic inversions shown as arcs of different colors along outer ring: red: C8 inversion (red); Glutamate Dehydrogenase (GluDH) inversion (green); RAMP/paREP inversion (blue). Positions of paREP elements shown as ticks inside outer ring: paREP1 (red); paREP2b (blue); paREP7 (green). Positions of selected genes which are present in *P. oguniense* and missing in *P. arsenaticum* are shown in text inside outer ring: thiamine biosynthesis genes (ThiW and ThiC); CRISPR Cassette(CAS); cobalamin cluster; CO dehydrogenase(COdh); and the aerobic cytochrome clusters(Cyto-c). Aligned regions smaller than 500 nucleotides have been removed for clarity.

The majority of the *P. oguniense* genome is structurally syntenic to the genome of *P. arsenaticum,* and genes found in both species show an average of approximately 96% nucleotide identity. The *P. oguniense* genome is approximately 15% larger than *P. arsenaticum,* with the former encoding 536 more (2835 - 2299) open reading frames (ORFs) predicted to be genes. Vast stretches of sequence space are syntenic between the two species (Figure 2, regions in blue), broken by relatively few regions that appear to arise from either gene loss in *P. arsenaticum* or genomic expansion in *P. oguniense*, possibly a result of the numerous paREP elements present in these genomes (Figure 2). These repetitive regions are difficult to assemble, and some are putative transposons (PaREP2b, for example).

We can identify specific genes and gene clusters that are present in *P. oguniense* but are missing in *P. arsenaticum*. Notably, the cobalamin synthetic cluster and two thiamine synthetic genes (ThiW and ThiC) are absent in *P. arsenaticum*. The terminal cytochrome cluster associated with aerobic respiration [35] is also absent in *P. arsenaticum* as expected from an obligate anaerobe. Among the 16 largest deletions in *P. arsenaticum* (relative to *P. oguniense*), four are associated with paREP2 genes, six with paREP1/8, and one with paREP6 (Table 5).

## Conclusion

Genomic sequencing and assembly of *Pyrobaculum oguniense* has yielded a complete genome and an extra-chromosomal element. The main chromosome is largely syntenic to *Pyrobaculum arsenaticum* and contains a number of gene clusters that are absent in that species. This is of particular interest considering that these species were isolated on opposite sides of the Eurasian continent; *P. oguniense* was isolated in Japan, while *P. arsenaticum* was isolated in an arsenic-rich anaerobic pool in Italy.

The synteny that has been retained between the genomes of *P. oguniense* and *P. arsenaticum* allows a close examination of gene gain or loss events in the genetic history of these two species. *P. arsenaticum* is missing the gene clusters that support cobalamin and thiamine synthesis, and it is missing the aerobic cytochrome cluster. Given that *P. oguniense* and the next closest member in the clade, *P. aerophilum,* have both retained these capabilities; the most parsimonious explanation is gene loss in *P. arsenaticum*. Because these genes are located at disparate positions in the *P. oguniense* genome, it would further appear that these losses are the result of multiple events in the evolutionary history of *P. arsenaticum*.

Within this genome, 145 non-coding RNA genes are described. These include a single operon encoding 16S and 23S ribosomal RNA, the associated 5S rRNA, the 7S signal recognition particle(SRP), and the RNase P RNA. There are 47 annotated tRNA genes, plus a single tRNA pseudogene. Also included are 83 predicted C/D box sRNA genes and nine additional H/ACA-like sRNA, each of which has been transcriptionally validated [31]. The non-coding RNA content of the *P. oguniense* genome has become the most extensively annotated among crenarchaeal genomes to date.

The use of a not-quite-clonal cell population for DNA isolation, coupled with ultra-deep sequencing has provided a view of three major inversions that are each present in over 17% of the sample population. The boundaries of one of these inversions are defined by an inverted repeat encoding a duplication of glutamate dehydrogenase (GluDH). Notably, this duplication appears to be present in each of the currently sequenced *Pyrobaculum* members, suggesting that those genomes may also host similar inversions. A second inversion has at its termini another inverted duplication, encoding a gene associated with one of the paREP members and a CRISPR-associated gene. It remains unclear if these common structural variants impart a physiological advantage, and if so, how the variation provides utility to its host. Based on our expanded genome diversity observations, we suggest that avoiding the use of a strictly clonal population for sequencing purposes can provide a significant benefit to understanding both the biology of the host and a clearer understanding of the genome dynamics of the species.
